# Network Systems Pharmacology-Based Mechanism Study on the Beneficial Effects of Vitamin D against Psychosis in Alzheimer’s Disease

**DOI:** 10.1038/s41598-020-63021-8

**Published:** 2020-04-09

**Authors:** Peihao Fan, Xiguang Qi, Robert A. Sweet, Lirong Wang

**Affiliations:** 1 Department of Pharmaceutical Sciences, Computational Chemical Genomics Screening Center, University of Pittsburgh School of Pharmacy, Pittsburgh, USA; 2 Department of Neurology, University of Pittsburgh School of Medicine, Pittsburgh, USA; 3 Department of Psychiatry, University of Pittsburgh School of Medicine, Pittsburgh, USA

**Keywords:** Systems biology, Computational biology and bioinformatics, Computational neuroscience

## Abstract

Alzheimer’s disease (AD) is a chronic neurodegenerative disease with significant financial costs and negative impacts on quality of life. Psychotic symptoms, i.e., the presence of delusions and/or hallucinations, is a frequent complication of AD. About 50% of AD patients will develop psychotic symptoms (AD with Psychosis, or AD + P) and these patients will experience an even more rapid cognitive decline than AD patients without psychosis (AD-P). In a previous analysis on medication records of 776 AD patients, we had shown that use of Vitamin D was associated with delayed time to psychosis in AD patients and Vitamin D was used more by AD-P than AD + P patients. To explore the potential molecular mechanism behind our findings, we applied systems pharmacology approaches to investigate the crosstalk between AD and psychosis. Specifically, we built protein-protein interaction (PPI) networks with proteins encoded by AD- and psychosis-related genes and Vitamin D-perturbed genes. Using network analysis we identified several high-impact genes, including NOTCH4, COMT, CACNA1C and DRD3 which are related to calcium homeostasis. The new findings highlight the key role of calcium-related signaling pathways in AD + P development and may provide a new direction and facilitate hypothesis generation for future drug development.

## Introduction

Alzheimer’s disease (AD) is a chronic neurodegenerative disease commonly seen in the aging population, and the presence of AD is responsible for a significant decrease in the quality of life^1^. It is estimated that the cost of AD is $604 billion worldwide and will triple in Year 2050^2^. Genetic factors are the second strongest risk factor for AD following age^3^. However, multiple environmental factors can be associated with the development of AD including medication usage^4^.

Psychosis, defined by the occurrence of delusions and/or hallucinations, is observed as a common complication of AD and patients with dementia. Literature reports that approximately 50% of AD patients will have psychotic symptoms (AD with psychosis, or AD + P) in the following years^5^. AD + P patients are considered as a subgroup of patients who have more severe symptoms, and are associated with more significant cognitive impairment and a quicker cognitive decline^6^. AD + P is also associated with higher rates of co-occurring agitation^7^, aggression^7,8^, depression^9,10^, mortality^11^, functional impairment^12^, and increased caregiver burden^13^ than AD patients without psychosis (AD-P).

Psychosis in AD often represents a significant additional drain on top of the baseline disease burden. AD + P patients are found to have a more rapid decline in the cognitive and memory functions, and the presence of psychosis can significantly increase the difficulty for caregivers. The symptoms of psychosis can cause considerable distress to patients^14–16^. In addition to these effects, AD + P is a marker for other adverse outcomes in AD. The most associated behavior disturbances with AD + P are agitation and aggression;^7,8,17^ depressive symptoms are also increased in AD + P patients^9,10^.

In a previous study, we have compared the frequency of medication usage among AD + P and AD-P patients and conducted survival analysis on time to psychosis for AD patients to identify drugs with beneficial effects^18^. The results of our analysis revealed a significant association between Vitamin D use and delayed onset of psychotic symptoms. In addition, through the analysis of gene expression data, we found that AD- and/or psychosis-related genes were enriched in the list of genes most perturbed by Vitamin D. This observation provides us with a novel direction for the mechanism study of AD and psychosis, and may inspire the development of drugs to prevent or treat psychosis in AD.

The role of Vitamin D in neurodegenerative diseases has been reported by many researchers. Six of the nine case-control studies found significant between-group differences illustrated by lower serum concentrations of 25-hydroxyvitamin D, a metabolite of Vitamin D_3_, in AD cases compared to control groups^19–25^. Thus, Vitamin D Insufficiency is considered as a risk factor for AD. However, Vitamin D’s beneficial effect against AD + P was freshly discovered and its mechanism may provide a unique viewpoint in preventing and treating AD + P.

Network approaches have been used in predicting and identifying the disease genes in multiple studies and some of the results have been verified^26,27^. It is suggested that, in the viewpoint of network biology, drug targets tend to locate at the transition area from the essential hubs, e.g. proteins interacting with more partner proteins, to redundant peripheral nodes^28^, e.g. proteins interacting with fewer partner proteins. The rationale behind this is a balance of toxicity and efficacy regarding the potential influence of the targets on cellular function.

The aim of this study is to explore potential molecular mechanisms that underlie the beneficial effects of Vitamin D on reducing psychosis symptoms in AD patients and to identify potential drug targets for AD + P prevention or treatment by applying systems pharmacology approaches on analyzing their protein-protein interaction networks.

## Method and Material

### Gene dataset collection and pathway mapping

A network that includes both AD- and psychosis-related proteins were constructed and analyzed in order to study the crosstalk between them. AD- and psychosis-related genes were collected from multiple literatures and databases, including MetaCore from Clarivate Analytics (https://portal.genego.com/), GWAS Catalog for Genome Wide Association Studies (GWAS) (https://www.ebi.ac.uk/GWAS/home)^29^ and BaseSpace Correlation Engine (https://www.illumina.com/index-d.html)^30^. These gene names were then converted to protein names by batch search function in the UniProt database. The criteria for including genes in our study are described in supplementary material. Vitamin D-perturbed genes and antipsychotics-perturbed genes were collected from BaseSpace Correlation Engine (https://www.illumina.com/index-d.html)^30^. Both down- and up-regulated genes were included into our analysis.

Signaling pathways for AD and psychosis were acquired from KEGG (http://www.genome.jp/kegg/)^31^ and PANTHER Classification System (http://pantherdb.org/)^32^.

### Network analysis with centrality measures

In the following network analysis studies, we incorporated protein-protein interaction (PPI) data from STRING (https://string-db.org/)^33^ and the Online predicted human interaction database (OPHID) (http://ophid.utoronto.ca/ophidv2.204/index.jsp)^34^. The PPI network was constructed and analyzed with python package networkx (https://networkx.github.io/)^35^. The interaction network was shown in the molecular action view with the medium confidence level (>0.4)^36^. The network containing AD-related proteins (AD network) and the network containing psychosis-related proteins (Psychosis network) were joined to form a combined network (AD-psychosis combined network) for further study. PPI networks containing Vitamin D-perturbed proteins (Vitamin D network) and antipsychotics-perturbed proteins (Antipsychotics network) are also generated respectively.

Centrality measures of the nodes were introduced in network analysis to describe how the information will spread through the network. Two different kinds of centralities were included: Degree Centrality and Betweenness Centrality. Degree Centrality, as the most simple and direct, describes the number of connections of a particular node regardless of the direction and weight of the edges. Betweenness Centrality, as the centrality of control, represents the frequency at which a point occurs on the geodesic (shortest paths) that connect pairs of nodes. In another word, it quantifies how many times a particular node acts as a bridge linking two ends of the network.

Networks were processed and plotted with python package networkx^35^ and Gephi^37^. The centrality of nodes in the network was calculated based on the built-in algorithm of networkx^35^. In detail, the degree centrality values were normalized by dividing by the maximum possible degree in a simple graph n-1 where n is the number of nodes in a network. The Betweenness centrality algorithm is from Ulkrik Brandes^38–41^.

In order to minimize the bias caused by the number of studies associated with different proteins, we use Betweenness centrality as our primary indicator in this study to learn more on the nodes’ position in the network’s structure, rather than the degree centrality of the nodes in the network.

Network analysis methods with centrality measures will first be examined with psychosis-related genes and known antipsychotics-perturbed targets. In order to do that, a combined network of psychosis network and antipsychotic network is constructed and the centrality measures are calculated as mentioned above. The connectivity parameters of known antipsychotic targets are examined to determine if they possess a significantly higher value.

To find sub-networks (communities) having different biological functions, community detection was further conducted in the combined network. The algorithm used for community detection was based on the Greedy Modularity Maximization method^42,43^. It begins with each node in its own community and joins the pair of communities that most increases modularity until no such pair exists.

### Triple-focusing network approaches: identification of potential novel targets

Network analysis was further used to study a joint AD-psychosis-Vitamin D network in order to find potential drug targets for AD + P. The rationale of this approach was that the ideal potential targets should be in the overlapping part of PPI networks of AD, psychosis and Vitamin D because the function of the potential targets can modulate the crosstalk between AD and psychosis and can also be regulated by Vitamin D through the Vitamin D receptor which is a transcriptional factor modulating gene expression. Thus, after constructing the AD-psychosis combined network and Vitamin D network, we overlapped them to explore the connectivity of these three parts and the roles of the triple-overlapped proteins.

Triple-focusing on AD-, psychosis-related and Vitamin D-perturbed proteins can help us reduce the artificial bias caused by the different amount of studies of those proteins, and also limit the potential side effects caused by targeting those very well-studied proteins which are usually located at the essential hubs. The identified small groups of proteins will have the potential to act as targets for Vitamin D to modulate AD- and psychosis-related networks.

## Results

### Method verification with psychosis-related PPI network and antipsychotics-perturbed genes

Psychosis-related and antipsychotics-perturbed PPI networks are used to validate the network analysis methods we proposed. Characteristics of these two PPI networks and the combined network are shown below (Table 1). Five genes, DRD2, DRD3, HTR2A, OPRD1 and HTR7, are found shared by psychosis network and antipsychotics network.

**Table 1:** Characteristics of Antipsychotics- and Psychosis-related PPI networks.

Network Name	Node Number	Edge Number	Average Degree Centrality	Average Betweenness Centrality
Antipsychotics	89	419	0.106	0.0157
Psychosis	486	1409	0.0119	0.00642
Psychosis-antipsychotics Combined Network	570	1825	0.0112	0.00563

The centrality measures of nodes in the psychosis and antipsychotics are calculated and the top ten nodes sorted by Betweenness values were shown in Table 2. As we expected, DRD2 and HTR2A, two major targets for current antipsychotics, were ranked as the first two proteins in our combined network when measured by Betweenness Centrality. If ranked by Degree centrality, ALB and FOS, two well-studied proteins, will have higher priority than HTR2A. The result revealed the great potential for proteins with a high Betweenness centrality being drug targets and provided a solid support for the method we proposed. Thus, the network analysis methods were applied to AD- and psychosis-related PPI networks.

**Table 2:** Overview of net-influencers for top ten proteins (named by their genes) in combined network of psychosis and antipsychotics sorted by Betweenness centrality.

Gene Name	Degree Centrality	Betweenness Centrality
DRD2	0.0703	0.1433
HTR2A	0.058	0.0731
GRIA1	0.0615	0.0698
ALB	0.0633	0.0677
CACNA1C	0.0545	0.0513
FOS	0.0615	0.05
SYNE1	0.0246	0.0488
GRIN2A	0.0545	0.0485
FYN	0.0545	0.0432
KIT	0.0422	0.0368

### The AD-psychosis combined PPI network

In order to acquire a better understanding of the connection between AD and psychosis, and to further explore the potential drug targets suggested by the previous analysis, a combined PPI network of AD and psychosis was generated. One thousand and sixty-one AD-related genes and 15,691 PPIs of their protein products together with 483 psychosis-related genes and 1,361 psychosis-related PPIs were collected as the basis of our network. Among all the proteins collected, 90 proteins were shared by both AD and psychosis, including proteins encoded by SEMA3A, TUSC3, RPN2, AMBRA1, BECN1, CACNA1C, SGK1, ADAM10, GRIN2A, FYN, ANK3, TBXAS1, EFNA5, POLN, CHRNA3, NOTCH4, GRIA1, NTRK3, IQGAP2, RELN, NOS1, GPC6, TCF7L2, TCF4, MGLL, DRD3, CHRNA2, PAK2, CTNNA2, COL25A1, COL12A1, AGER, KIF26B, PPP2R2B, TEK, KALRN, PRKG1, KSR2, COLGALT2, MEIS1, SHISA9, ZKSCAN4, PTPRG, NKAPL, CTNNA3, PDE4B, HFE, MSR1, CSMD1, COMT, APBA1, IMMP2L, ELAVL4, LRRTM4, CDH13, ZNF804A, PBRM1, LRRN2, TEP1, STXBP5L, FHIT, SYNGAP1, ZSCAN31, TENM4, ABCB1, PLCL1, RBFOX1, FSTL5, SORCS3, NKAIN2, GLIS3, NXN, MAGI2, MEGF10, MPP6, TSPAN18, FRMD4B, MTHFD1L, TMTC1, LIN28B, UXS1, BICC1, ATXN7L1, EYS, GRAMD1B, TSPAN2, ENOX1, TMEM132D, CR1 and PCNX. The AD-psychosis combined network has 1,454 nodes and 16,948 PPIs. Characteristics of the combined network were most similar to those of AD network due to the disparity of the node numbers in AD- and psychosis-related PPI networks (Table 3).

**Table 3:** Characteristics of AD- and Psychosis-related PPI networks.

Network Name	Node Number	Edge Number	Average Degree Centrality	Average Betweenness Centrality
AD	1061	15691	0.0279	0.00167
Psychosis	486	1409	0.0119	0.00642
AD-psychosis Combined Network	1456	16989	0.0160	0.00158

**Figure 1:**
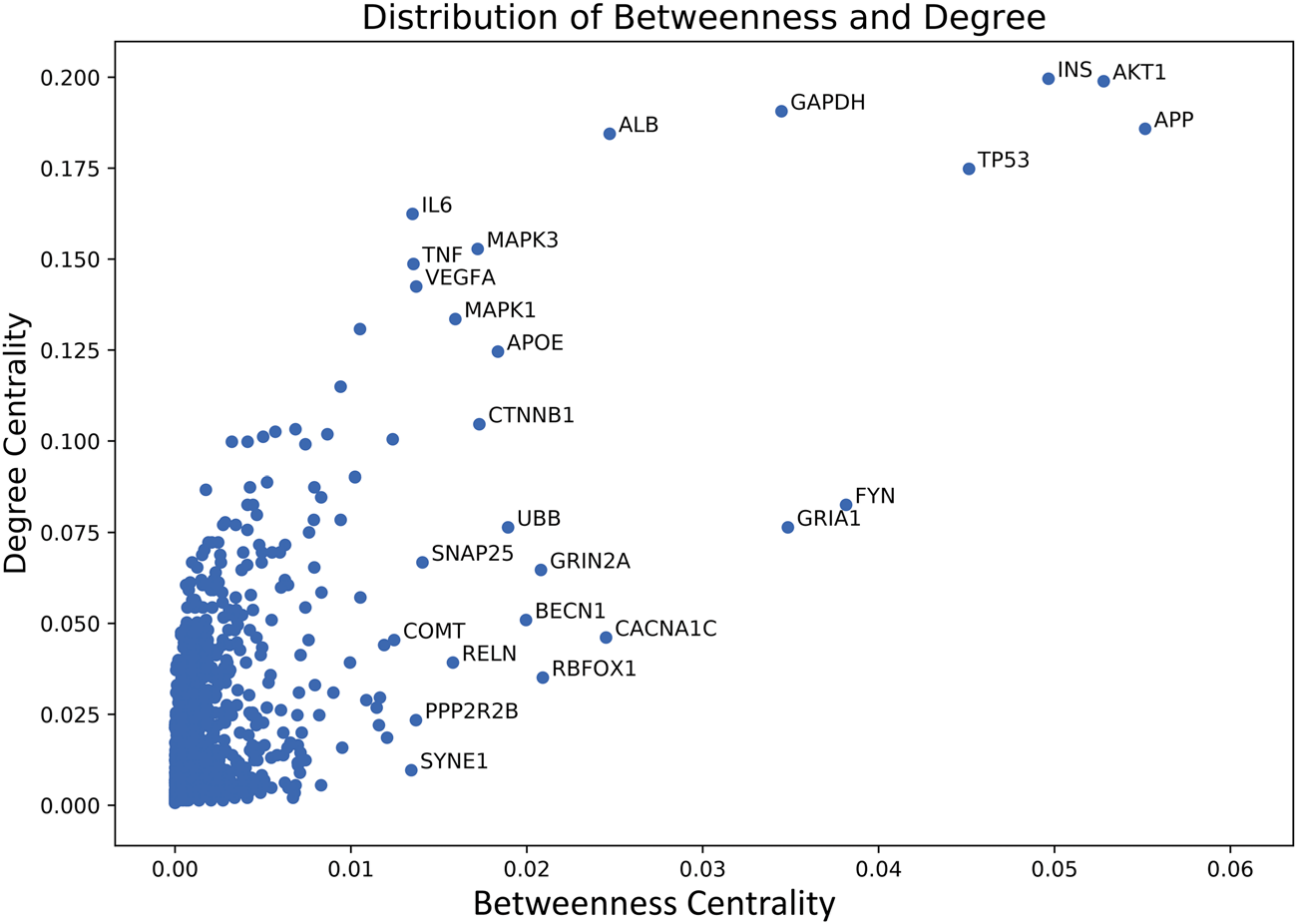
Distribution of Degree centrality and Betweenness centrality of nodes in the combined AD-psychosis PPI network. Most of the nodes have very low Degree centrality and Betweenness centrality while a very small group of nodes, like the top 10 nodes, possess very high centrality compared to others. This phenomenon suggests that the information flow within the network is controlled and regulated by the small group of nodes to a great extent.

Top 10 net-influencers in the combined network are shown in Table 3 based on their Degree and Betweenness centralities respectively. It is not surprising that the 3 centralities overlapped with each other substantially, since they all measure the importance of the nodes in the whole network from different angles, and it is apparent that the top 10 nodes do have very higher values when compared with the average value, 10-fold ratio at least. A better view is provided in Fig. 1 showing that only a few nodes take position at the upper-right corner. This phenomenon suggests that though there are 1,454 of nodes in the network, a small group of nodes, such as the top 10 nodes shown in the table, are extremely connected and play a critical role in the signaling process and information flow within the network.

**Figure 2:**
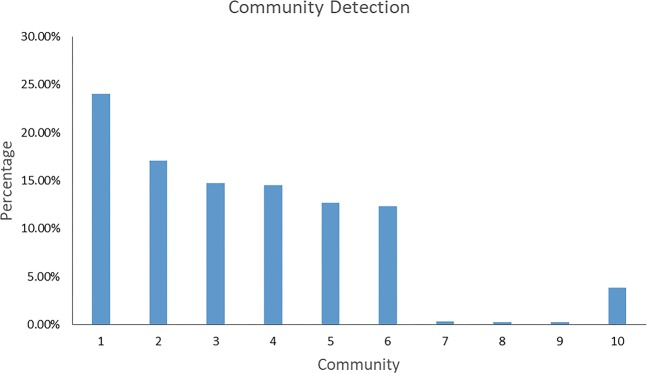
Overview of community detection. Seven meaningful communities are detected, and targets distributions are shown in the figure. These communities are constructed with similar targets amounts and can be the representatives for different biological functions involved.

**Figure 3:**
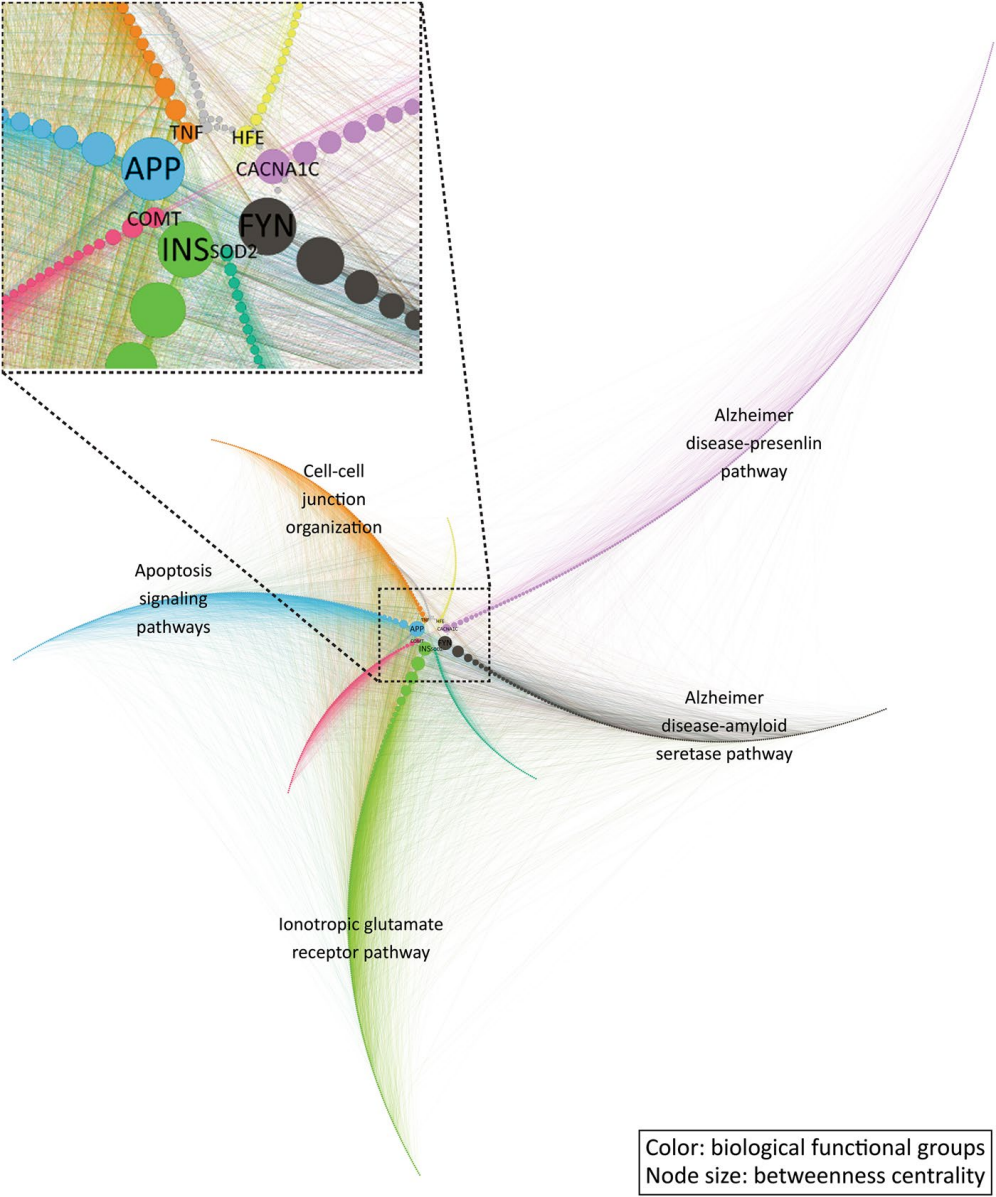
Overview of community interaction. Community interactions incorporated with the Betweenness centrality data of nodes and the functional annotations of the communities. The node size represents the Betweenness centrality of nodes. The high impact nodes, nodes with high Betweenness centrality, are evenly distributed to communities and function as the main gateway for information exchange and interactions. The architecture of the combined network is a big system formed by several sub-networks (communities) that connect with each other through a small hub, and most of the proteins in the network work mostly with the proteins within their communities. Figure generated with Gephi (https://gephi.org/) version 0.9.2.

After identifying the critical proteins in the network, the function of these proteins is our interest. We conducted pathway enrichment analysis to identify the underlying pathways participated by those proteins and therefore to establish a connection between proteins and their biological functions. Firstly, ten communities were detected as relatively separated components of the network (Fig. 2). Among the 10 detected communities, 7 communities, excluding 7, 8 and 9, contain enough nodes to be biologically meaningful. When sorting the network based on the community and the nodes’ Betweenness, every community has one or a few nodes that possess a much higher Betweenness value and those nodes serve as the portal connecting the community to the other parts of the network (Fig. 3). Among the top 10 proteins we mentioned above (Table 4), APP, FYN, and INS are distributed into different communities as the leading nodes, which further illustrates the importance of these nodes in the combined network. These detected communities represent different biological pathways participating in the development of psychosis in AD. Secondly, protein-pathway mapping was conducted by comparing the proteins in the same community against the proteins in the pathways from online databases like KEGG.

**Table 4:** Overview of top net-influencers in the AD-psychosis combined PPI network.

Gene (Degree Centrality)	Gene (Betweenness Centrality)
INS(0.200)	APP(0.0552)
AKT1(0.199)	AKT1(0.0528)
GAPDH(0.191)	INS(0.0497)
APP(0.186)	TP53(0.0451)
ALB(0.184)	FYN(0.0382)
TP53(0.175)	GRIA1(0.0348)
IL6(0.162)	GAPDH(0.0345)
MAPK3(0.153)	ALB(0.0247)
TNF(0.149)	CACNA1C(0.0245)
VEGFA(0.142)	RBFOX1(0.0209)

The protein-pathway mapping returned a list of pathways associated with these 7 communities evidenced with very low False Discovery Rate (FDR) adjusted p-values, meaning that the proteins in these communities are highly accordant with proteins in these pathways recorded in the database. Pathways closely related to AD and neurological disorders (Table 5) were enriched in the list, including the Huntington disease pathway, Alzheimer’s disease-presenilin pathway, p53 pathway and Alzheimer’s disease-amyloid secretase pathway.

**Table 5:** Results of protein-pathway mapping in the communities.

Community	Pathways (Pathway ID)	P-value
Community 1	FAS signaling pathway (P00020)	<0.001
Community 1	Ras Pathway (P04393)	<0.001
Community 1	PDGF signaling pathway (P00047)	<0.001
Community 1	Angiotensin II-stimulated signaling through G proteins and beta-arrestin (P05911)	<0.001
Community 1	Interleukin signaling pathway (P00036)	0.00236
Community 1	Wnt signaling pathway (P00057)	0.00121
Community 1	Huntington disease (P00029)	0.00367
Community 1	p53 pathway (P00059)	0.00459
Community 1	Alzheimer disease-presenilin pathway (P00004)	0.00138
Community 1	p38 MAPK pathway (P05918)	0.0132
Community 1	Parkinson disease (P00049)	0.0135
Community 1	Integrin signaling pathway (P00034)	0.0294
Community 2	Ionotropic glutamate receptor pathway (P00037)	<0.001
Community 2	Muscarinic acetylcholine receptor 1 and 3 signaling pathway (P00042)	<0.001
Community 2	5HT1 type receptor-mediated signaling pathway (P04373)	<0.001
Community 2	Enkephalin release (P05913)	<0.001
Community 2	Synaptic vesicle trafficking (P05734)	<0.001
Community 2	Heterotrimeric G-protein signaling pathway-Gq alpha and Go alpha mediated pathway (P00027)	<0.001
Community 2	Metabotropic glutamate receptor group II pathway (P00040)	<0.001
Community 2	Endothelin signaling pathway (P00019)	0.00296
Community 2	Opioid proopiomelanocortin pathway (P05917)	0.00136
Community 3	Alzheimer disease-amyloid secretase pathway (P00003)	<0.001
Community 4	Apoptosis signaling pathway (P00006)	<0.001
Community 5	Plasminogen activating cascade (P00050)	<0.001
Community 5	Cholesterol biosynthesis (P00014)	0.0223
Community 6	Cadherin signaling pathway (P00012)	0.0494
Community 10	Cell-cell junction organization (R-HSA-421270)	0.00992
Community 10	Nectin/Necl trans heterodimerization (R-HSA-420597)	0.0177
Community 10	Cell junction organization (R-HSA-446728)	0.0275

**Figure 4:**
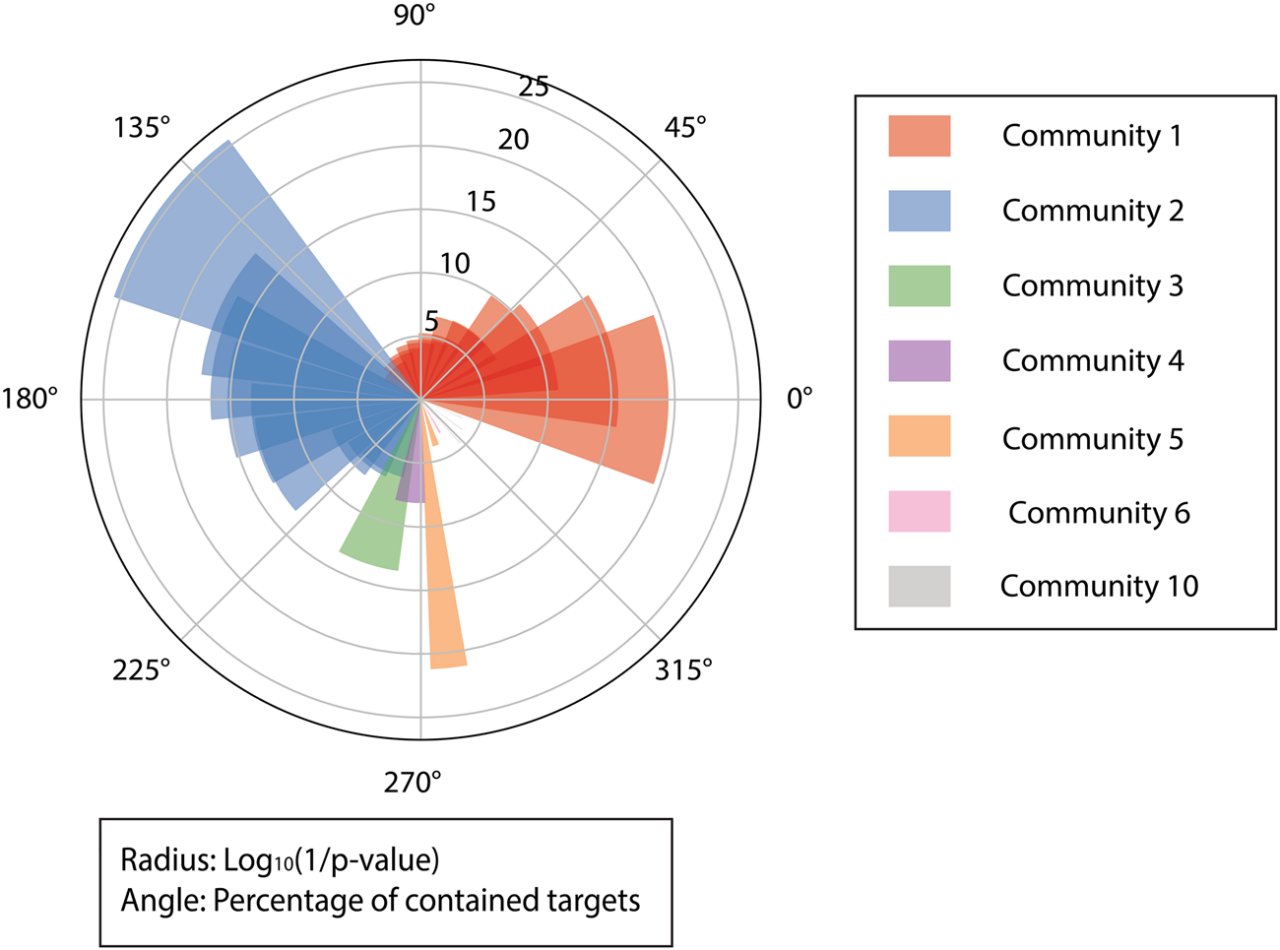
Distribution of proteins in the communities and p-values for protein-pathway mapping results. The radius represents the log_10_ (1/p-value) of a mapping, and a higher bar has a smaller p-value. The angle of the bar represents the percentage of proteins contained in the mapped community. Shades in same color indicate multiple pathway-matchings of one community. P-value is calculated by Fisher’s exact test and all terms are adjusted by Benjamini-Hochberg FDR. Figure generated with matplotlib (https://matplotlib.org/) version 3.1.3^63^.

Figure 4 provided a more direct overview of the results of protein-pathway mapping. Community 1 and community 2 were mapped to multiple pathways with high credibility. It is fairly understandable because these two communities contain the largest amounts of targets and may result in mismatches.

### Overlapping proteins between AD network and Psychosis network

Since the objective of this study is to study the development of psychosis in AD, we focused on the overlapping proteins between AD and psychosis. The net-influence parameters of the 90 overlapped proteins are shown in Table 6. Most proteins in the overlapping part possess Betweenness values above average which further supports their bridging role in the networks.

**Table 6:** Overview of net-influencers for overlapping proteins (named by their genes) between AD network and Psychosis network.

Gene Name	Degree Centrality	Betweenness Centrality
SEMA3A	0.0220	0.0046
TUSC3	0.0048	0.0025
RPN2	0.0048	0.0019
AMBRA1	0.0055	0.0002
BECN1	0.0509	0.020
CACNA1C	0.0461	0.0245
SGK1	0.033	0.008
ADAM10	0.0571	0.0105
GRIN2A	0.0647	0.0208
FYN	0.0826	0.0382
ANK3	0.0268	0.0115
TBXAS1	0.0083	0.0021
EFNA5	0.0255	0.0042
POLN	0.0055	0.0026
CHRNA3	0.0117	0.0012
NOTCH4	0.020	0.0072
GRIA1	0.0764	0.0348
NTRK3	0.0248	0.007
IQGAP2	0.0055	0.0038
RELN	0.0392	0.0158
NOS1	0.044	0.0119
GPC6	0.0145	0.0071
TCF7L2	0.0296	0.0117
TCF4	0.020	0.0062
MGLL	0.0172	0.0066
DRD3	0.0482	0.0043
CHRNA2	0.0145	0.0007
PAK2	0.0241	0.0046
CTNNA2	0.022	0.0116
COL25A1	0.0124	0.0035
COL12A1	0.011	0.0015
AGER	0.0303	0.0042
KIF26B	0.0055	0.0007
PPP2R2B	0.0234	0.0137
TEK	0.0262	0.0060
KALRN	0.0289	0.0109
PRKG1	0.0310	0.0070
KSR2	0.0103	0.0022
COLGALT2	0.0076	0.0009
MEIS1	0.0117	0.0020
SHISA9	0.0096	0.0006
ZKSCAN4	0.0055	0.0069
PTPRG	0.0151	0.0021
NKAPL	0.0055	0.0043
CTNNA3	0.0124	0.0024
PDE4B	0.02	0.0037
HFE	0.0186	0.0121
MSR1	0.0248	0.0082
CSMD1	0.0138	0.0058
COMT	0.0454	0.0125
APBA1	0.0248	0.0044
IMMP2L	0.0124	0.0047
ELAVL4	0.0165	0.0051
LRRTM4	0.0062	0.0006
CDH13	0.0110	0.0023
ZNF804A	0.0151	0.0048
PBRM1	0.0096	0.0026
LRRN2	0.0028	0.0009
TEP1	0.0062	0.0050
STXBP5L	0.0124	0.0074
FHIT	0.0165	0.0044
SYNGAP1	0.0193	0.0013
ZSCAN31	0.0034	0.0003
TENM4	0.0076	0.0017
ABCB1	0.0310	0.009
PLCL1	0.0028	0.0002
RBFOX1	0.0351	0.0209
FSTL5	0.0048	0.0019
SORCS3	0.0055	0.0045
NKAIN2	0.0041	0.0003
GLIS3	0.0069	0.0031
NXN	0.0083	0.0017
MAGI2	0.0145	0.0044
MEGF10	0.0034	0.0003
MPP6	0.0055	0.0003
TSPAN18	0.0028	0.0004
FRMD4B	0.0021	0.0002
MTHFD1L	0.0103	0.0006
TMTC1	0.0034	0.0001
LIN28B	0.0034	0.0012
UXS1	0.0048	0.0064
BICC1	0.0055	0.0083
ATXN7L1	0.0048	0.0019
EYS	0.0069	0.0024
GRAMD1B	0.0028	0.0027
TSPAN2	0.0048	0.0018
ENOX1	0.0014	0
TMEM132D	0.0048	0.0055
CR1	0.0124	0.0004
PCNX	0.0014	0.0001

**Figure 5:**
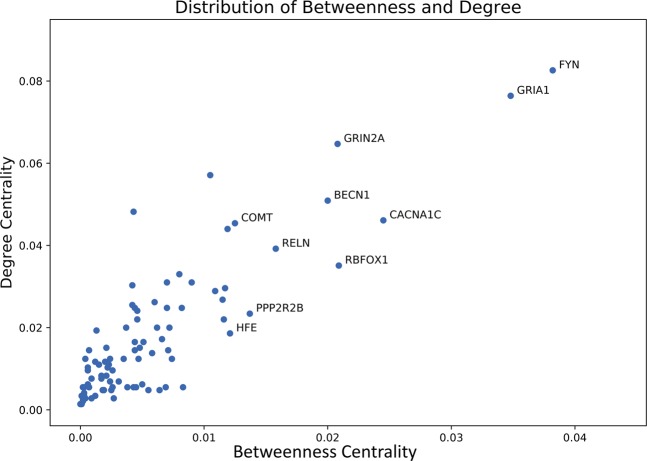
Distribution of Degree centrality and Betweenness centrality of overlapping proteins between AD network and psychosis network. FYN and GRIA1, as members of the top 10 targets, possess a far larger Degree centrality and Betweenness centrality among the overlapping proteins. Figure generated with matplotlib (https://matplotlib.org/) version 3.1.3^63^.

Figure 5 shows the distribution of connectivity parameters of overlapping proteins. Even in the overlapping part of the network, the average Betweenness centrality remains relatively low and only a few nodes, like FYN and GRIA1, possess a much higher connectivity than other nodes. The distribution of Betweenness follows the same pattern as the whole network suggesting that even though 90 targets are found overlapped between psychosis and AD, only a few of them are the “bridges” for the transferring of information.

### Exploration of Vitamin D’s beneficial effect through a triple-focusing approach

In our previously published paper, Vitamin D was identified as a promising medication with a significant association with decreased occurrences and delayed onset of AD + P^18^. Therefore, we examined the relationship between the Vitamin D network and the AD-psychosis combined network. In total, 89 targets and 344 PPIs were collected in the Vitamin D network (Table 7). Among the 89 proteins, twenty-one are shared with the AD-psychosis combined network. Net influence parameters are calculated for these 21 targets and sorted by their Betweenness centrality values (Table 8).

**Table 7:** Characteristics of Vitamin D network.

Network Name	Node Number	Edge Number	Average Degree Centrality	Average Betweenness Centrality
Vitamin D	89	344	0.0869	0.018

**Table 8:** Overview of top net-influencers ranked by Betweenness values for overlapping proteins (named by their genes) between AD-psychosis combined network and Vitamin D network.

Gene Name	Degree Centrality	Betweenness Centrality
CACNA1C	0.0461	0.0245
COMT	0.0454	0.0125
NOTCH4	0.02	0.0072
DRD3	0.0482	0.0043
CD36	0.022	0.0024
EGR1	0.0619	0.0022
CCL2	0.0867	0.0018
DLX5	0.0062	0.0010
CYP1A1	0.0227	0.0008
A2M	0.0358	0.0006
VDR	0.0282	0.0006
TGFB2	0.0296	0.0006
TIMP3	0.0268	0.0006
CD14	0.0227	0.0006
CYP19A1	0.0296	0.0004
NME1	0.0227	0.0003
HSD11B1	0.0131	0.0002
MMP12	0.0227	0.0002
AMBRA1	0.0055	0.0002
ALOX15	0.0117	0.0001
GIG25	0.0145	0.0001

After sorting by the Betweenness centrality, CACNA1C, COMT, NOTCH4 and DRD3 are ranked as the top four proteins. Their positions in the overlapping part of the combined network allow them to function more as a bridge to link different components of the network, which also suggests a therapeutic potential for AD + P. Therefore, these four proteins gained our special interest. One interesting thing is, when we look back at Fig. 1, these four targets fell into the middle distribution of values for Degree centrality and Betweenness centrality, which matched the conclusion that drug targets tend to be positioned at the transition area in a biological network^28^.

**Figure 6:**
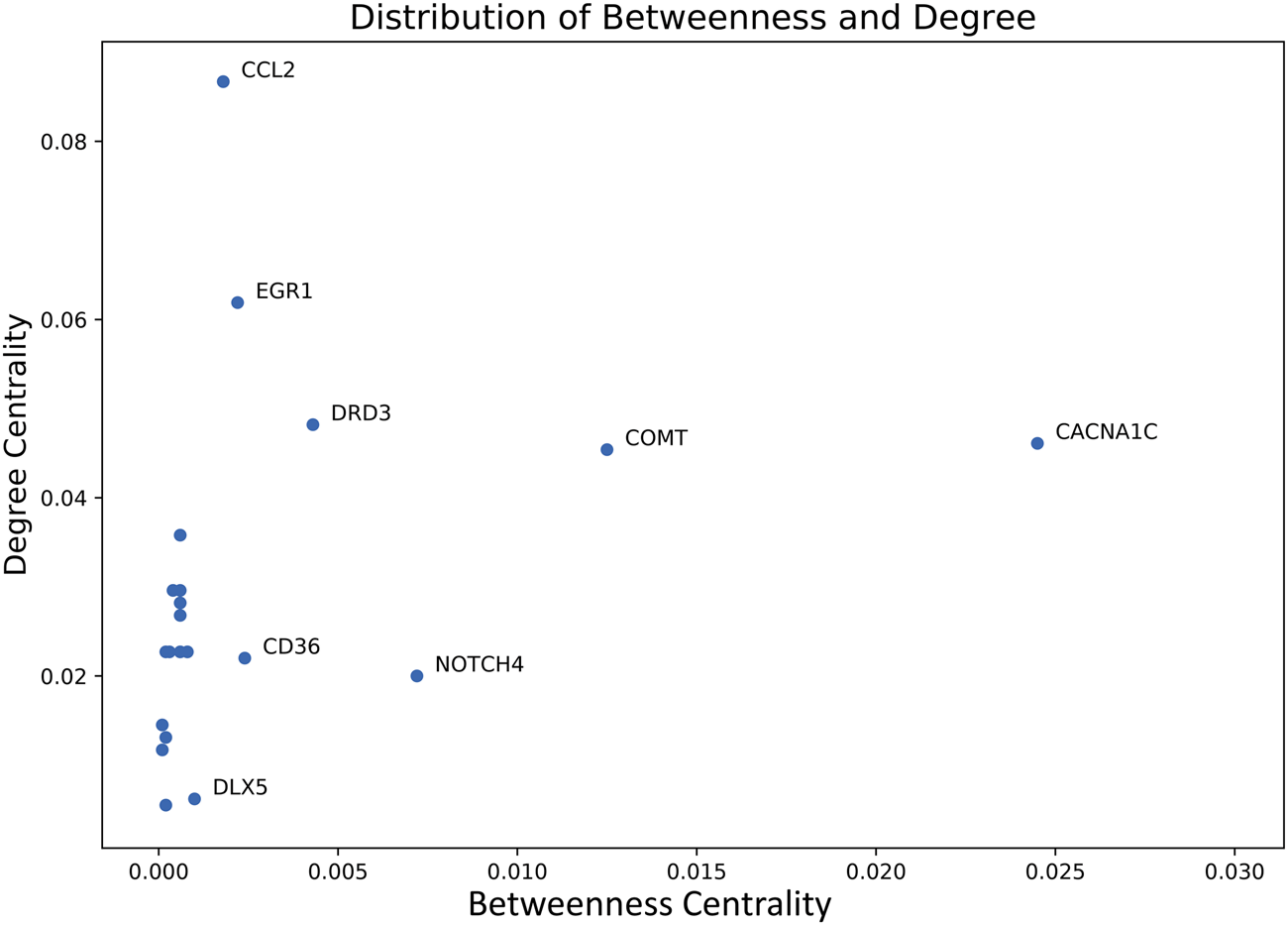
Distribution of Degree centrality and Betweenness centrality of overlapping proteins between AD-psychosis combined network and Vitamin D network. Overlapping proteins between AD-psychosis combined network and Vitamin D network follows the same pattern as the whole networks. Some nodes like CACNA1C, COMT, NOTCH4 and DRD3 possess much higher Betweenness centrality values than the average value of the network. Figure generated with matplotlib (https://matplotlib.org/) version 3.1.3^63^.

Figure 6 shows the distribution of connectivity parameters of overlapping proteins between AD-psychosis combined network and Vitamin D network. The 21 overlapped nodes followed the distribution of the whole combined network and revealed several proteins with outstanding Betweenness centrality values. These proteins will tend to act as the “bridges” in communicating AD-, psychosis-related network and Vitamin D perturbed network and thus the potential explanation of the beneficial effects of Vitamin D against AD + P.

## Discussion

The network analysis based on the protein-protein interaction data have presented us four potential targets encoded by genes *CACNA1C, NOTCH4, COMT and DRD3* that may account for the beneficial effects of Vitamin D against AD + P. These four potential targets all possess high enough connectivity to alter the crosstalk between AD and psychosis. In addition, variants in *CACNA1C, NOTCH4* and *COMT* had been reported to be associated with schizophrenia in GWAS studies^44–46^. Among them, the function of *CACNA1C, NOTCH4* and *COMT* were reported to be closely associated with calcium homeostasis^47–53^ which can be further associated with Vitamin D’s effect. Similarly, after the activation of DRD3 by dopamine, the Gβγ complex is released and can interact directly with voltage-gated calcium channels^54,55^. Except for NOTCH4, all are targeted by marketed drugs for different indications. Interestingly, DRD3 is one of the primary targets for antipsychotics in treating psychotic symptoms in schizophrenia or other neurological disorders^56,57^. Alternative splicing of DRD3 in the transcription process may result in encoding different isoforms that are functionally impaired^58^. Although limited, there is some support for targeting DRD3 in the treatment of AD + P^59,60^. However, verification of DRD3 or the other of these four potential targets for AD + P will require additional studies.

The beneficial effect of Vitamin D against AD have been widely reported. The protective effect of Vitamin D can be executed by reducing the oxidative and nitrosative damage caused by elevated levels of nitric oxide (NO) and inducible nitric oxide synthase (iNOS) in nerve cells^61^. There is also evidence suggesting an overlap between the disruptions of vitamin D pathways with amyloid pathology which can partially explain the protective role of Vitamin D in AD^62^. However, this study is the first study to explore the mechanism of Vitamin D’s beneficial effect against AD + P. In this study, the triple-focusing approach we use can help minimize the bias caused amount of studies and restrain our scope at Vitamin D related potential targets.

There are limitations in this study. The PPI networks were constructed based on the protein-protein interaction data extracted from databases, thus they are limited by the amount and availability of data in the databases. Also, there is no direction information attached with most PPIs which means our PPI networks are undirected. Therefore, centrality measures can be biased by the direction information in actual situations.

## Conclusion

In this study, various approaches of network analysis are incorporated with systems pharmacology to provide a systematic overview on the crosstalk among AD, psychosis and Vitamin D at the molecular level. The triple-focusing network method helps us explore the designated mechanisms for Vitamin D’s effects on AD + P and a potential explanation is provided: Vitamin D regulates several genes encoding proteins that play critical roles in the overlapping part of the AD-psychosis combined network, which allow them maximally influence the signaling and information transfer process. In other words, proteins with high net-influence that localize at the triple-overlapped part of the AD, psychosis and Vitamin D network, like *CACNA1C*, *COMT*, *NOTCH4* and *DRD3*, possess the ability to play an important role in the crosstalk among AD and psychosis by delivering Vitamin D’s effect to the transiting hub connecting the AD network and psychosis network. Thus, the four identified potential targets can be crucial in explaining Vitamin D’s beneficial effect against AD + P. To conclude, the results from this study provided a possible explanation of the beneficial effect of Vitamin D against AD + P and presented a new direction for drug development with four potential novel targets.

## Supplementary information

Supplementary Information.

## Data Availability

Full gene lists used in this study can be found in the supplementary material. The detailed PPI interaction data and full net-influence parameters list are available on request.
