# Supplementary material for: Network Systems Pharmacology-Based Mechanism Study on the Beneficial Effects of Vitamin D against Psychosis in Alzheimer’s Disease
Source: Sci Rep. 2020 Apr 9;10:6136. doi: 10.1038/s41598-020-63021-8 (PMC7145835; doi:10.1038/s41598-020-63021-8)
Supplement: Supplementary file 1 — Supplementary Information. [file 41598_2020_63021_MOESM1_ESM.pdf]

Supplementary Materials

Network Systems Pharmacology-Based Mechanism Study on the Beneficial Effects of Vitamin D against Psychosis in Alzheimer's Disease

Peihao Fan[1], MS Xiguang Qi[1], BS Robert A. Sweet[2, 3, 4*], MD Lirong Wang[1*], PhD

[1]Department of Pharmaceutical Sciences, Computational Chemical Genomics Screening, University of Pittsburgh School of Pharmacy, Pittsburgh; [2]Department of Neurology, University of Pittsburgh School of Medicine, Pittsburgh; [3]Department of Psychiatry, University of Pittsburgh School of Medicine, Pittsburgh; [4]VISN 4 Mental Illness Research, Education and Clinical Center, VA Pittsburgh Healthcare System, Pittsburgh.

*Corresponding Authors:

Robert A Sweet, MD

UPMC Endowed Professor in Psychiatric Neuroscience

Professor of Neurology and Clinical and Translational Science

3811 O'Hara Street

Pittsburgh, PA 15213

Email: sweetra@upmc.edu

Phone: 412-624-0064

Lirong Wang, PhD

Assistant Professor

Computational Chemical Genomics Screening Center

Department of Pharmaceutical Sciences/School of Pharmacy

University of Pittsburgh

528 Salk Hall, 3501 Terrace Street

Pittsburgh, PA 15213

Email: liw30@pitt.edu

Phone: 412-624-8118

Inclusion criteria for genes

Due to the variety of information sources, genes were carefully selected from different types of data including RNA and miRNA expression, SNPs identified through GWAS, copy number variations (CNVs), and mutation data. 1) reported in a primary GWAS analysis, defined as array-based genotyping and analysis of 100,000+ pre-QC SNPs selected to tag variation across the genome and without regard to gene content; 2) SNP-trait p-value <1.0 x 10^{-5} in the overall population. The threshold of 1.0 x 10^{-5} was chosen rather than the stricter one for genome-wide association of 5.0 x 10^{-8} [1,2] to include more targets potentially related to AD and psychosis to generate a more complete structure of networks[3,4].

Alzheimer's disease-related genes

HLADRB1	EIF4EBP1	A2M	LRP1	ABCA1	ABCA2	ABCA7
ABCC2	ABCG1	ACAD8	ACE	ACHE	CHRM2	ACTA2
ACTL8	ADAMTS1	ADAM10	ADAM12	ADAR	AGBL4	AGTR1
AGTR2	AKT1	ALDH2	ALOX12	ALOX15	ALOX5	AMBRA1
RUNX1	ANKH	ANKRD2	ANKRD6	ANO3	APEX1	APH1A
APH1B	APLP2	APOA1	APOC1	APOC1P1	APOC2	APOC4
APOD	APOE	LRP8	APOF	LPA	APOM	APP
NAE1	APPBP2	PRNP	ARHGAP30	ARPP19	SRRT	ARSB
ASAH1	ATAD2	ATF2	ATF4	ATP10B	ATP13A5	ATP8A1
ATXN7L1	ADCY2	ACAN	ADAMTS4	AGRN	SERPINA1	ACTR1A
SNCA	IAPP	AR	ANK3	ANXA13	TNFSF10	AQP1
ARSA	AXIN1	B4GALT3	B9D2	BACE1	BACE2	PBRM1
SMARCD3	ADGRB3	BAP1	DDX39B	BCAM	BCAS3	BCR
BCYRN1	BCHE	BDNF	BDNF-AS	BICC1	BIN1	PRUNE2
SLC25A14	BMP5	ASIC2	BRD3	BCL3	BCL2L2	BECN1
ADRB1	CTNNB1	BLMH	BRCA1	C10orf55	C14orf177	CLUHP3
MTHFD1L	STUM	C1R	MROH8	PP2D1	C5AR1	C6orf48
CACNA1C	CACNA2D4	CALB1	CAMK1D	CARD8	NEDD9	CBLC
CBS	CCDC83	CACNG4	CCL2	CCR3	CCR5	CD14
CR2	CD2AP	CD33	CD40	CD40LG	CD8A	CD86
CDK1	CDK5	CDK5R1	CDK5R2	CDON	CEACAM16	CEP164
CETP	CFTR	CHD6	STUB1	CHAT	CLCN6	CLEC16A
CLIC5	CLPTM1	CNGB1	CNR2	CNTN5	COG1	VPS13B
COL18A1	COMT	COPA	COX6C	PTGS1	PTGS2	COX10
CR1	CR1L	CREB1	CRHR1	LINC02210-CRHR1	CRP	CSF1
CSTF2T	CTGF	CUX2	TET1	CYP17A1	CYP19A1	CYP1A1
CYP27C1	CYP46A1	CAMK1	CAMK2A	CAMK2G	RCAN1	S100A9
S100A12	CAPN1	CAST	CSNK1D	CSNK2A2	CASP2	CASP3
CASP4	CASP8	CASP9	CNTNAP2	CTNNA3	CTSB	CTSD
CTSS	ADAP1	CP	CH25H	CHGA	CLU	F5
F8	F13A1	COL19A1	CRH	CSMD1	CUBN	CCNC
CCND1	CST3	DAPK1	DAXX	ASAP2	DEDD	DEFB122
GLUD1	DIO1	DIO2	DIP2C	DIRAS2	DISC1	PARK7
DKK1	DLD	DLGAP1	POLB	DNAJC12	DNMBP	DONSON
KCNIP3	TSC22D3	DVL1	DYNC1I1	DYRK1A	CTNND2	DRD1
DRD3	DRD4	DBN1	DNM2	E2F1	EBF3	ECE1
EDEM2	EFCAB7	EGR1	ELOVL6	ENO1	ENO2	ENTPD7
ERAP1	ERG	KCNH6	MAPK3	MAPK1	ESR1	ESR2
CELF2	EYA4	EYS	POMK	EPHA1	EPHA5	EFNA5

EPOR	ERBB4	FAAH	FAF1	OTULIN	CALHM1	MINDY1
FAM89B	UBD	FDPS	FGF1	FHIT	ENOX1	INO80D
BASP1-AS1	C5orf64	FMNL2	FMO2	FSTL5	FTO	FNTA
FASLG	FAS	FCER1G	FCGR2B	APBB1	APBB2	APBB3
AHSG	FLOT1	FYN	GNAO1	GNB3	GAPDH	GAPDHS
GCNT1	GAB2	GALNT17	GBP2	GABBR1	GCFC2	PLEKHG5
GFAP	GGA1	SLC1A3	GBA	GLUL	GLS2	GOLM1
RXFP3	ADGRG1	GRAMD1B	GRK2	GSK3A	GSK3B	GSTA4
GSTO1	GSTO2	GSTP1	GSTT1	GAL	GALP	SNCG
GSN	GHRL	GRIA1	GRIA4	GP6	GPC1	FABP3
HSD17B10	HECW1	HHEX	HLA-A	HFE	HLX-AS1	HLX
HMGCR	HMGB1	HMGCS2	ARHGAP45	HOOK3	HSD11B1	HSPB8
HSPA1B	HSPA2	CWC15	HTR1A	HTR2A	HTR2C	HTR6
ZWINT	HMOX1	ELAVL4	RNR2	HTT	IGFBP2	IGFBP3
IGFBP6	ICAM1	ICAM4	IDE	IFNG	IGF1	IGF1R
IGF2	IGF2R	IL1A	IL1B	IL10	IL18	IL2
IL33	IL6	IL1RN	IL6R	CXCR2	IMMP2L	CXCL10
ITPR1	IQCK	IREB2	TCF4	ITGAM	ITM2B	IVD
INS	INSR	IQCE	JAK2	F11R	MAPK8IP1	KLHDC9
KCNAB2	KCNH7	KCNMA1-AS1	KCNQ3	CKB	WASHC4	KANSL1
FAM214A	FAM120B	WWC1	KIF13B	KIF26B	KLC1	KIF11
KLK10	KLK6	KLK7	SLC4A1AP	KCNC4	L3MBTL4	LAMA1
LAMC1	LDLR	LHCGR	LHFPL6	LINC00466	LINC00475	LINC00836
LINC01122	LIPA	STK11	LMOD3	LOC101929570	LOC145845	RBM15-AS1
EMBP1	LPL	LRAT	LRP6	PPP1R37	LRRK2	LRRN2
LRRTM3	LRRTM4	LRTM2	LUZP2	LMNA	LCK	MAGI1
MAGI2	MAL2	MAOA	MAOB	MAPT-AS1	MAPT-IT1	MARCH10
MARK1	MAT1A	MBOAT1	SLC16A7	ABCB1	MEF2A	MEIS1
MAP2K1	MAP2K2	MEX3A	MGAT5B	MGST1	MIF	CCL3
CCL4	CCL4L1	MMP12	MMP8	MPP7	MPZL1	LRPAP1
MRE11	MROH7-TTC4	MS4A4A	MSI1	MSR1	STK24	MTHFR
ND1	MTR	STXBP1	MYH13	MYH7B	KCNMA1	MTNR1A
MS4A6A	MYOCD	MYOF	MYO10	NACA	QPRT	NALCN
ART3	NAT1	NAT2	NAV2	NCAM1	SLC8A1	NME1
NME1-NME2	NME2	NDRG2	NDUFS2	NEFH	NEFL	NGFR
TMEM147	NINJ2	NIPBL	NKAIN2	NKAPL	NOTCH1	NOTCH4
NPAS2	NPC2	NPFFR2	NPY	NQO1	GRIN1	GRIN2A
GRIN2B	SLC11A1	SLC11A2	NQO2	ITGB3BP	NTF3	NUMB

NECTIN2	NECTIN4	MME	NRG1	NCAN	NCSTN	NIT1
NKPD1	NXN	DLST	OGG1	OLIG2	OLR1	OPA1
OSGEP	OTUD7A	POU2F1	SELP	P2RY2	PADI2	SERPINE1
PARP1	PAXIP1	PCDH11X	PCGF5	PCMTD1	PCSK9	PDE4B
PDE9A	PDZD7	MPO	PFDN2	PGBD1	PTGES2	PTGDS
PICALM	PITRM1	PRKACA	PRKCA	EIF2AK2	PLA1A	PLA2G2A
PLAC4	PLAT	PLAU	PLCE1	PLXDC2	PNMT	POLN
POMC	POMT1	PON1	PON2	PON3	POTEA	PPARD
PPARG	PCK1	PPID	PPOX	PPP1R11	PPP1R1C	PPP1R3B
PPP2R2B	PRDX1	PRDX2	PRDX3	APBB1IP	PRKAR2A	PRKAR2B
PRND	PRIMA1	DLG4	PSMB9	PSMC4	PTEN	HACD1
PTPRA	PURA	GART	PVR	ALDH18A1	PAWR	PCNX1
PSENEN	HSPG2	PIN1	PLG	PSEN1	PSEN2	PCSK1N
PDYN	GRN	PREP	PRKG1	PTK2B	RSPO2	RACK1
RAD51B	AGER	GAPVD1	RASSF4	RDH13	RGS4	RPN2
ROR1	PLPPR1	RTN4R	RAB6A	RELB	RTN4	S100B
SAMSN1	SAR1A	ZFYVE9	SBSN	SCARA3	SCN2A	SERPINA3
SERPINF2	SLC6A4	SREK1IP1	SHISA9	ST6GAL1	SLC18A3	SLC1A2
SLC1A7	SLC4A2	SLC5A12	SLC6A17	SMAD3	NCOR2	SNAP25
SNCA-AS1	SNORD52	SNX10	SNX3	SOAT1	SOD1	SOD2
SORCS2	SORL1	SOS2	SP1	SGPL1	SCARB1	SIGMAR1
ZSCAN26	SSSCA1	SUPV3L1	SVIL	SYP	STH	SEC24C
CHGB	SEMA3A	SEPT1	SEPT3	SEPT7	SQSTM1	SIRT1
SST	GH1	SORCS1	SORCS3	STMN1	STXBP5L	SYN1
SYNPR	SYT1	STX8	ZNF365	TRIOBP	TARDBP	TAS2R60
TBP	TCF7L2	TDRD10	TEAD1	TEP1	TFAM	TFCP2
TFEB	TGFB1	TEK	TIMP1	TIMP2	TIMP3	TLK1
TLR4	TLR9	TM2D1	TMEM132D	TMEM147-AS1	TMEM177	TMED10
TNFRSF1A	TNFRSF1B	TNF	LTA	TNK1	TOMM40	TPH1
TPPP	THRA	TREM2	TRIM15	TRPC4AP	TSNAX-DISC1	TSPAN18
TSTD1	TTC27	TUSC3	TXNRD2	MAPT	TSPAN2	THOP1
TIAM1	KAT5	TMEM132C	TMTC1	TCN1	TCN2	TF
TTR	NTRK1	NTRK2	NTRK3	TTC4	TSC2	U2AF1L4
UBB	UBE2D1	UBE2Q1	UBXN4	UCHL1	UFC1	UGCG
UMAD1	UNC13C	USF1	USP21	USP35	UXS1	UBQLN1
UFSP1	MPP6	VDR	VEGFA	ZNF197	VSNL1	HPCAL1
MAVS	VLDLR	WDFY4	WT1	WEE1	APBA1	APBA2
XRCC1	XYLT1	TRAK2	ZBP1	ZFYVE19	ZNF155	ZNF224
ZNF225	ZKSCAN4	ZNF320	ZSCAN31	ZDHHC7	ZNF720	ZNF804A
ZC3H3	SLC30A1	SLC30A4	SLC30A6	ZYX	ABL1	RAF1

RAPGEF4	EEF2	EEF2K	EIF2S1	EIF4E	NOS3	MIR4713
MIR4761	MIR6843	MIR6886	MIR7846	NOS2	MTOR	TRNQ
CHRNA2	CHRNA3	CHRNA4	CHRNA6	CHRFAM7A	CHRNA7	CHRNB2
CHRNB4	NOS1	RELN	CDKN1B	TP53	RPS6KB1	TP73
ABCC13	AD10	AD11	AD12	AD13	AD14	AD15
AD16	AD5	AD6	AD7	AD8	AD9	ADORA2A
AGBL4-IT1	ALB	AMIGO2	APCS	APLP1	APOC4-APOC2	AQP4
ARMCX5-GPRASP2	ATP5F1A	AZIN1-AS1	BACH1	BHLHB9	CASP1	CASP6
CASP7	CCL11	CCL4L2	CCL5	CCL7	CD36	CFAP410
CHAF1B	CNR1	COL18A1-AS1	CRYAB	CXCR3	CYP2D7	CYP7B1
DAGLA	EPHA1-AS1	ERBB2	ERBB3	GCG	GHRLOS	GNAS
GPR55	GRIA2	GRIA3	HCRT	HNRNPA2B 1	HP	IL12A
IL12B	KALRN	KLK8	LINC00211	LINC00972	LINC01081	LINC01266
LINC01492	LINC01501	LINC02254	LINC02516	LOC100130 587	LOC100287 329	LOC100379 224
LOC100996 288	LOC101927 502	LOC101928 418	LOC101928 651	LOC101928 961	LOC102724 297	LOC414300
LOC541472	LOC643387	LOC646506	LRRC15	MELTF	MEOX2	MGLL
MMP2	MMP9	MS	MS4A4E	MUC22	NAPEPLD	OAT
P4HA3-AS1	PAK1	PDE8B	PLUT	PRDX6	PRKXP1	RABGAP1L
RNF146	SERPINF1	SLC18A2	SLC6A3	SNCB	SSSCA1-AS1	SYNPR-AS1
TNFAIP1	TRPV1	ANKRD22	LINC00624	RAD23BP2 - SEMA3A	MECOM	MCPH1-AS1
AKR7A3	IRAK1BP1	SCAPER	CEP63	SEC24B-AS1	TENM4	TMCO4
NACAP6 - LINC02150	DYSF - RPS20P10	DLC1	TMEM94	PLCL1	GPR180	LINC00626 - SUMO1P2
BAALC-AS1, BAALC	CCZ1B - OR7E39P	PCSK6	RF00438 - R3HDM2P2	CFAP74	CCDC112 - CTNNA1P1	CTNND2 - RNU6-679P
FAM240B	SLMAP	AOX1	UNC93B4	CDH1	IQGAP2	RASSF8
CEP295NL, TIMP2	FAM19A5	KLHL36 - USP10	PIFO	IGSF23	SELENOO	CDCA7L - RAPGEF5
BNIP3P13 - ZNF90	SYNGAP1	SESTD1	TAS2R5	CSNK2A1 - TCF15	C9orf152 - TXN	FAT3
RDX	ABCA8	STEAP3 - C2orf76	NEK10	ERO1A - PSMC6	RNU7-188P - SEM1	SEC24B
GTF2H3	PTPRS - ZNRF4	RASSF5	COL25A1	HMCN1	NAALADL2	RN7SKP120 - TUSC1
RNA5SP169 - LINC02273	KSR2	MDGA2 - MIR548Y	OSER1-DT - GDAP1L1	JPH3	AQP4-AS1	DNAH6

HDAC9	SHANK2	UGT1A10, UGT1A8	TBXAS1	KIFC3 - CNGB1	LINC02103 - RNU6-909P	TSNAX-DISC1, DISC1
LINC01725	PAX2	PROX1-AS1	RN7SKP168 - ZFYVE9P2	ATP8A2P3, RNF6	EDAR - RF00017	EPC2 - RNU2-9P
	APOC1 - APOC1P1	MTCYBP27 - RNU6-976P	SIGLEC22P - CD33	BIN1 - NIFKP9	MRPL58 - RNU6-362P	FBXL7
CACNA2D3	ADAMTS9-AS2	DCHS2	PUM3	HRK	SLC28A1	MLN - LINC01016
DYNLL1P4 - RBM19	PRRC2C	FMN2	CTNNA2	LIMS2	MOBP	STK32B
AFF1	ANKRD55	PGAM5P1	CAMK4	DMXL1	MEGF10	LINC01184
SAP30L	PLEKHG1	BZW2	CYCS	ELMO1	EXOC4	NCS1
PLPP4	SPON1	ARHGAP20	SLC4A8	CRADD	ANO4	GPC6
MYO16	CLMN	GABRG3	LIPC-AS1, ALDH1A2, LIPC	TNRC6A, LINC01567	VAT1L	SP6
CACNA1G	PPIAP59	TGM6	PARVB	RPS17P11 - MFSD4BP1	RPL3P11 - ATP5PDP3	CASC18
NDUFA12	FANCD2OS, FANCD2	RN7SL782P - RN7SKP122	IL19	NCKAP5 - RN7SKP93	CCDC85C	SDR9C7
NARS2	PKNOX2	LMOD3 - FRMD4B	SLC25A6P5 - LINC01505	LINC02098 - ETS1	OSBPL6	RNU6-248P - RNU6-261P
HNF4G - RNU2-54P	LAMP1	RBFOX1	ARVCF	UBXN11	HYI, SZT2	AHCYL1
RF00012 - NMNAT1P2	TGFB2	RNU4-77P - KCNK1	ITSN2	OTOF	MSH2	LINC01185
RAB1A - RF00090	SPRED2	LINC01965	RN7SKP141 - SMC4P1	COL4A4	RANP7 - SALL4P5	RPEP2 - HMGB3P12
C3orf67	PTPRG	RN7SL271P - UBE2Q2P9	LINC02008	TFP1	RNU6-637P - TERC	SNRPCP13 - ENPP7P11
RNU6-412P - RAC1P2	BANK1 - SLC39A8	SETD7	SH3RF1	LINC02268	RNU6-381P - FGF10	F2R - F2RL1
WDR41	MEF2C-AS1	RPS17P2 - LINC02214	RF00019 - ZCCHC10	LARS	G3BP1	KDM1B
CDKAL1	DST	COL12A1	LINC02532 - CD24	SGK1	SNX9	TULP4
PHF14	MTDHP1 - ZNF117	PQLC1P1 - EEF1A1P28	DLX5	LYPLA1P1 - IQUB	INSIG1	NKAIN3
SGK3	PPP1R42	FZD6 - CTHRC1	PDCD1LG2	LINC01243 - MTATP6P30	ATP5MFP3 - RFC5P1	LINC00476
FOXE1 - TRMO	MTND3P4 - ARL2BPP7	PNPLA7	WAC	RPL34P19 - RN7SL825P	C10orf71	RNU6-478P - MARK2P15
MICAL2	TRIM51CP	AHNAK	CCDC89	AICDA	OVCH1-AS1	RNU7-106P - OTOGL

TPTE2P3	RABEPKP1 - DACH1	ARF4P4 - LINC00377	FARP1	LINC00343	FAM181A	LINC02304 - LINC02325
HERC2	TRPM1	THSD4	AGBL1	TMC5	SDR42E2	CDH13
ALOX12-AS1	COX10-AS1	MPP3 - CD300LG	DCAF7	TCAM1P	CDH19 - RNU6-1037P	ZNF813
CST1 - CSTP2	RPS2P1 - ASIP	LINC01271 - RN7SL636P	SYNJ1	TEX33 - TST	RAB20	PDS5B
SPSB1	BDH1, BDH1	ADARB2	TOP1	LIN28B	C2orf83	TIAM2
RPL7P19 - ETF1	GOLIM4	RNF165	SLC25A48	COLGALT2	ABCB11	ARAP2
IRF2	AKAP9	DSCAML1	IL34	LINC01838 - ZNF30-AS1	SLC25A5P3 - VSTM2A	TRIQK
LINC01508	RSPO4	KAZN	CCDC134	MS4A4E - MS4A4A	PICALM - RNU6-560P	CLU - SCARA3
ZNF292	CDC42EP3	UTS2B	PAK2	ADCY8	RNF219-AS1	RORA-AS1
SLC44A5	NME9	CCRL2	MMP3 - MMP12	NFU1P1 - MYRIP	NRXN3	TLN2
BMPER	SLC8A1-AS1	SLC9A9	EFR3A	LINC02343	SLC24A4	ZFP3 - ZNF232
CEACAM22 P	RF00285 - BCL3	MS4A4A - MS4A6E	BCAM - NECTIN2	TOMM40 - APOE	APOC4	APOC2
CLASRP	GEMIN7-AS1	PPP1R37	INPP5D	CD2AP - ADGRF2	ADGRF2	PILRA
AGFG2	AP4M1	CASTOR3	PMS2P1	EPHX2	GULOP	PSMC3 - RAPSN
SPI1	FERMT2	BCKDK	ZNF232	CNN2	CEACAM20	APOC4
CLPTM1 - RELB	GEMIN7	MARK4, PPP1R37	GEMIN7-AS1	EXOC3L2, MARK4	MARK4, EXOC3L2	CSTF1 - CASS4
HLA-DRB1 - HLA-DQA1	GPR141, EPDR1	CELF1	CLU	CR1; CR1	MTCO3P30	SLC2A9
RNU1-80P - TNRC6C	RIMBP2	RNU6-276P - EXOC1L	KRT18P16 - LINC01170	SUCLG2	GMNC - OSTN	GLIS3
PFDN1 - HBEGF	NFIC	ZAP70	PUS1	MAP2K5	ANGPT4	ARIH1
LINC01098	HIGD1AP3 - MSX2	FAM83E	RNU4ATAC8 P - LRRIQ3	KCNN3	HSPA8P9 - CLDN18	RN7SL691P
IL21-AS1	SERINC5 - KRT18P45	CDC42SE2	SH2D4B	FRMD4A	IRF6	PMS2CL
THSD7A - TMEM106B	LINC02210-CRHR1	ARL17B	WNT3	HID1-AS1 - CDR2L	MACROD2 - PPIAP17	DAPL1
PDE1A	CADM2	FBXO40	OFCC1	PEX6	SORD	LINC01684
ABI3	PLCG2	RHBDF1	ATP5F1C	BSG	C11orf65, ATM	TECTA
TREM2 - TREML2	NDUFAF6	AP2A2	IGHV2-70 - IGHV3-71	TRIP4	ZCWPW1	CASS4

Psychosis-related genes

HTR2A	DRD2	HTR7	GRB10	SLAMF1
NFKB1	ACKR1	AS1	ZNF618	COMT
GRM7	AGER	SLITRK1	ADGRL2	GSAP
TMEM26	AQP8	HS3ST3A1	SCN8A	RGS6
FAM43A	NRG3	AC087071.1	CAMKMT	SLC3A1
STX8	ZNF473	NSG1	PLA2G4A	DOCK1
FAR2P1	ST5	HS3ST4	COMT	MKNK1
KIT	TBX1	CNTN4	LIPC	AC079950.1
CCDC60	MSRA	CEACAM21	AC108734.4	TCF4
BCL9	TMEM245	COL25A1	SGCD	LINC00499
ADCK1	C3orf38	CCDC122	PHF20	ENOX1
PTPN6	EFNA5	DMAC1	AC012254.2	NFS1
PLCB1	COL26A1	IQGAP2	DLGAP2	MECR
CTNNA2	STXBP5L	TENM4	EYS	AC009468.1
BMPR1B	NRG2	TCHP	HS6ST1	CEP41
SLC14A2	COLGALT2	RGL1	CSMD1	NAV2
RICTOR	OSMR	AC009652.2	COL12A1	KHDRBS2
MPP4	TEP1	LINC01435	NFATC2	MEGF10
SPATA6L	PCDH7	CNTNAP5	TNIK	SPATA6L
NBEA	RTN1	RBFOX1	FANCA	RBKS
ZNF385B	GPM6A	TENM3	MIR583HG	ATXN7L1
FAM120A	MAD1L1	AC120114.1	CWC22	GRM3
LINC01725	CACNA1C	KLF12	AKAP6	APOPT1
AC104574.2	MSI2	ZEB2	RMND5A	FXR1
SLC39A8	BANK1	GRIA1	SLC17A3	POM121L2
ZSCAN31	CARMIL1	ABCB1	IMMP2L	AL161716.1
GLIS3	MRTFA	AKT3	FHIT	AC244033.2
SH3GL3	ZSWIM6	SHC4	AL672167.1	NTRK3
RALGAPA1	TMEM132D	CIR1	HCN1	ADCY1
NR3C2	SPATS2L	LPP	GABBR2	CNTN2
ZFHX3	AC092650.1	DST	ZDHHC2	KLF6
RPSAP52	PLCL1	EFR3B	ERCC8	XYLB
GRID1	DLG2	DNAJC11	CALN1	NOL4
MOSMO	ZDHHC8	RAB8B	PIK3C2A	SMG6
ZNF536	PRKD1	AL022476.1	EMX1	AL163541.1
GGNBP1	MPHOSPH9	SHISA6	TENM2	TBC1D29P
PCLO	ARHGAP40	INHBA	ZBTB7B	ADGRV1

NOS1	DOP1B	DNAH1	CACNA1D	LIMK2
KIF1BP	TBC1D5	TMEM182	CHRNA2	SFMBT1
MIR137HG	BCL11B	LILRP2	AL591368.1	EEFSEC
MAIP1	AC011306.1	OPCML	FRMD5	FAM86B3P
SCAPER	KYAT1	PTPRF	PSORS1C1	LIN28B
NAB2	ZNF664	NLGN1	SPG7	AC114763.1
AC008667.1	MIRLET7BHG	ROBO2	NTM	AL583808.1
NPAS3	LHFPL3	AC022784.1	DGKI	CDC25C
CYP26B1	QPCT	CACNA1I	SNAP91	SDCCAG8
ETF1	RIT1	AC091862.1	BTN3A1	AL662884.2
TSNARE1	STUM	LINC02219	EPN2	MAN2A1
LIMA1	DOP1A	SRR	LINC02267	DCC
PLCH2	FURIN	LEMD2	MAGI2	ASAP1
PTPRD	APBA1	ATP2A2	FAM214A	RUSC2
OSBPL10	TTC12	KALRN	TRPM6	MTHFD1L
OPRD1	NRGN	TSPAN2	CNNM2	ARHGEF26
ALMS1	AC021594.2	PRRG2	YPEL1	GNG7
ZBED4	MMP16	PCNX3	TCF20	AL035685.1
PPP1R16B	STK4	CLIP1	LRRTM4	HIST1H2APS4
PRKG1	SORCS3	CALB2	RHBDL3	ZNF804A
FOXP1	LINC02438	CMAHP	GUSBP2	TEK
SRPK2	DNAJA3	AP000688.2	STAG1	LINC00862
SATB2	ZFAND2B	PPP1R13B	NEU1	TMTC1
PCNX1	LINC00606	LINC01470	SLCO6A1	HFE
MSR1	EBLN3P	CACNB2	GLG1	LINC01539
ZCCHC14	PHACTR3	PTGIS	KIF21B	SCN9A
ZNF823	SP4	SOX5	GULOP	CMAHP
AL645941.2	CUX1	AC097634.4	IGSF9B	AC046136.1
MIR29B2CHG	NEGR1	TRAPPC3	CAPN2	LRRN2
ZCCHC17	VRK2	AC096570.1	SPHKAP	BCL11A
EPC2	TMEM178B	PPP2R3A	FRMD4B	KLHL29
DPYD	SLC9B2	SHISA9	AC008415.1	PTPRK
GRAMD1B	SNORC	POU6F2	FMN2	TTYH3
CHD2	AC090578.1	LINC01122	CPEB1	ANKRD23
FER	ALOX15P2	CACNB2	SKAP1	RTKN2
AL445623.2	MINDY2	DGKZ	TMCO5B	AC067752.1
CHRNA3	NFATC3	RPTOR	SPECC1	GID4
AP005203.1	PCBP3	THOC7	PSD3	THRB
ZDHHC20	PCDH9	FOXO3	AIG1	SYNGAP1

ADAMTSL3	HS6ST3	GPC6	ANKRD36	SNAPC3
SUFU	HECW2	AL391117.1	ATP2B2	PLCL2
ELAC2	YWHAE	GRIN2A	U91319.1	HCP5
ITPR3	JAM3	AP002851.1	AL138974.1	FOXO6
CENPM	PEPD	BNIP3L	PAK2	CLCN3
AC117377.1	POC1B	TACC2	LINC02551	LRP4
C12orf76	AC016866.1	NT5C2	LINC01360	AC009226.1
RN7SL100P	PCDH15	BICC1	AC024901.1	AC005906.2
AC002070.1	GPM6A	AL162726.3	KIAA0391	AL121694.1
AL358790.1	AP003174.1	MARK2	LETM2	AGO4
CRB1	NEURL1	CFAP58	MTUS2	ACTG1P22
CUL3	MGLL	GALNT10	LINC00301	GRIN2B
IQANK1	RALGPS1	GALNT2	DMTF1	EBNA1BP2
PDE4B	LINC01776	ELAVL4	AL138927.1	SLC45A1
LINC02549	CCDC192	AP003049.2	LINC01583	AC087564.1
CIB4	SLC1A1	AL390957.1	LINC01310	DNAJB5
SPOCK1	PIGO	LINC00303	AC116337.3	ZP1
AC093766.1	CNTNAP4	ZNF665	DKK3	RELN
AGBL1	ACSM1	TRAF3	PIK3C2G	HHAT
ZFYVE28	LINC01255	RORA	KIF26B	NLRC5
ARNTL	CDH13	FARSB	SGK1	RENBP
TBXAS1	SGCZ	AGAP1	FEZ1	ANK3
PLAA	ACSM3	NOTCH4	BRD1	FBXO11
ARHGAP31	NKAIN2	PRRC2A	SNX29	AC027458.1
ANO4	AC026167.1	VIPAS39	B9D1	ZFAND6
PDE5A	SLC47A2	RASGRF2	EVL	PRMT8
PTPN13	TCERG1L	CACNA1S	AC060809.1	LINC01505
SAMD4A	PLD1	SIGLEC15	CHCHD6	PHACTR2
CR1	AC005871.2	RNF135	POMT2	CTNNA3
PIM3	EGFEM1P	USP24	SLC5A10	AL117329.1
POLN	AF127577.4	AC104041.1	AC092957.1	AC091078.1
ZBBX	ADGRE3	OXR1	EIF3F	CWH43
AC007179.2	NRP1	MIR100HG	TUSC3	XKR6
CACNA1B	AL512506.3	OSCP1	PRKN	NSMCE2
AFF3	AC091114.1	PTPRT	DPP10	FAP
AC018767.3	AC011369.1	CR1L	LINC02223	HIBCH
FSTL5	VPS45	MIR4432HG	ZNF362	TDRP
PECR	AMBP	ARHGEF28	CALD1	AC008892.1
CA12	NXN	GVQW3	PRLR	SLC17A6

TOX	ZNF611	SERPINA1	AC114689.3	C1orf167
ARL3	AC040169.4	AC069234.1	GTF2IP7	EEPD1
SIPA1L2	SPAG16	COL21A1	RASGEF1B	Z82202.2
AC078923.1	LINC01320	PPP3R1	RASSF1	LINC02232
FYN	YWHAG	KIAA1217	CLSTN3	HIP1R
AF123462.1	RYR3	AC006305.1	RERE	TRANK1
PBRM1	TNXB	UBE2Q2P1	AC022031.2	ITIH3
HYKK	CARMIL1	HIST1H1PS1	NT5C2	MDK
AMBRA1	IMMP2L	MEF2C	FAM86B3P	SFXN5
STK19	KDM4C	ZNF365	OR5V1	ZKSCAN4
TRIM27	AL662860.1	TRIM26	COX11P1	ZBED9
SFTA2	AL121936.2	HCP5	FLOT1	AL138726.1
SLC17A4	NSUN6	SLC7A6	AC092167.1	ADAM10
SLC9B1	PPP2R2B	FAM184A	PRDM14	ZFPM2
FES	TAOK2	AC012322.1	MAU2	ARHGEF10L
CABLES1	IPO8	LMO7	LTN1	PCDH12
PLCB4	RPN2	STX2	ZNF740	C11orf21
CCDC102B	GIGYF1	TMPRSS5	KSR2	UXS1
ACTL7A	ATP6V1E2	PHF2P2	LSM1	EDIL3
LRP1B	AC008474.1	NKAPL	TSPAN18	MPC2
LSM1	ITIH1	SPTLC1	MSRA	COMMD10
LINC00243	AC069228.1	AL138889.2	LINC01478	GABBR1
AC091096.1	TCF7L2	AGBL4	PSORS1C1	AL139300.2
DOCK4	TTLL6	ST3GAL1	ADAMTS16	ZZEF1
LINC01592	AC000403.1	RFESD	SNED1	ZNF93
FTCDNL1	GIGYF2	LINC00637	TMEM219	R3HDM2
SCAF1	AC104162.2	NLGN4X	NDRG4	NSD3
GSDME	RAI1	PLA2G15	SLC4A10	FAM178B
FAM177A1	LINC00461	AC007570.1	ZKSCAN3	OR5V1
DDR1	NGEF	AL662884.1	HIST1H2BN	ZSCAN9
MAD1L1	MUCL3	MIR137HG	CNOT1	F2
BTN2A2	FGFR1	CYP2D7	TWF2	RFT1
MTCO3P1	PBX2	LINC00240	CHRNA5	BTN3A2
OR11A1	KDM3B	BTN2A1	TRIM10	MUC22
C2	PBX2	HCG20	EHMT2	ATAT1
SFTA2	AL645929.2	STT3A	PLCB2	PTPRG
ITIH4	LINC02033	C12orf65	THOC7	AL807742.1
PRPF3	KIF5C	AL392086.1	AC007221.2	CLEC17A
PALB2	MPP6	GLT8D1	AC010538.1	ANKS1B

SLC35F2	SYNE1	HDAC4	ZNRD1	ZNF615
AC090993.1	SEMA3A	MRM2	GRIK1	ZMIZ1
AP001267.5	AC104009.1	NOTCH4	DRD3	CACNA1C

Vitamin D-perturbed genes

VDR	CYP27B1	CYP27A1	CYP2R1	CYP24A1
CD14	ATF3	THBD	SPP1	SERPINA3
FOS	GEM	EFTUD1	IL8	G0S2
TREM1	FN1	CLMN	THBS1	MAPK13
LCN2	EDNRA	CXCL1	FBP1	EGR1
CYP26B1	SHE	IFIT1	STAC3	VCAN
DPP4	CYP51A1	A2M	CAMP	HBEGF
EDN1	S100A8	OSR2	SULT1C2	SLC22A3
GCLC	C15orf48	HOPX	TGFB2	CYP19A1
GPNMB	DDX27	INMT	TIMP3	CD36
KCNK3	NREP	PDK4	IBSP	FGF9
MYC	CCL2	DLX5	SEMA6B	SEMA3C
HAVCR1	ALOX15	S100A4	GZMB	TGM2
HSD11B1	BTG2	CTSH	CYP1A1	CILP
BHLHE40	CCND2	AFP	IGFBP1	PPP2CA
CRISPLD2	TNC	ZFP36	NME1	CCL22
FABP4	CYP3A4	SCD	ORM1	VNN1
CA2	RGN	COL3A1	DUSP5	MED1
TNFRSF11B	C20orf197	HMGCS1	PVALB	NR0B2
PHLDA1	BCAT1	MMP12	C3	ROCK2

Antipsychotics-perturbed genes

DRD2	ACE	CACNA1G	HRH1	ADRA1A
ADRA1B	HTR2A	HTR2B	DRD4	HTR1A
ADRA1D	SLC6A2	HTR3A	DRD1	HTR2C
OPRD1	SLC6A4	SLC18A2	CHRM2	CHRM3
CHRM5	DRD3	HTR7	ADRB1	CHRM4
CHRM1	HTR1D	HRH4	CALY	EGR1
FOS	ATF3	CYP1A1	CYP51A1	CDK1
HMGCS1	CTGF	SULT1A1	MEIS2	CCNA2
ALB	ONECUT1	HERPUD1	GDF15	CA3
RRM2	STAC3	DDX27	EGR2	PHLDA1
CYP2E1	CCNB2	DBP	PPP2CA	HAMP

MKI67	RGN	BHLHE40	NREP	PLAU
SCD	FAM111A	HMOX1	SLC22A8	JUN
GJB1	CDKN1A	ALDH1A1	GZMB	DDIT4
CSDC2	TAGLN	CXCL1	IFIT1	RSAD2
CHAC1	SOX11	ZNF354A	FGF9	IGFBP5
CTH	FABP1	INSIG1	HSPA1B	LTN1
EDNRA	CCL2	LOC1720	CYP1B1	ENPP2
KLF4	NR4A3	LOX	SPP1	NR1D2
PPP3CA	CEP104	LDLR	HOMER1	IDI1

1 Okada, Y. *et al.* Meta-analysis identifies multiple loci associated with kidney function–related traits in east Asian populations. *Nat. Genet.* **44**, 904 (2012).

2 Hu, Y. *et al.* GWAS of 89,283 individuals identifies genetic variants associated with self-reporting of being a morning person. *Nature communications* **7**, 10448 (2016).

3 Shi, Y. *et al.* A genome-wide association study identifies new susceptibility loci for non-cardia gastric cancer at 3q13. 31 and 5p13. 1. *Nat. Genet.* **43**, 1215 (2011).

4 Yatagai, Y. *et al.* D91 REMODELING OF THE BRONCHOVASCULAR UNIT AND LUNG DISEASE: Genome-Wide Association Study For Late-Onset Asthma Demonstrates Possible Association With A Gene Related To Diffuse Panbronchiolitis. *Am. J. Respir. Crit. Care Med.* **191**, 1 (2015).
